# Various sources of distraction during analogical reasoning

**DOI:** 10.3758/s13421-022-01285-3

**Published:** 2022-02-24

**Authors:** Hanna Kucwaj, Michał Ociepka, Adam Chuderski

**Affiliations:** Institute of Philosophy, Jagiellonian University, Grodzka 52, 31-044 Krakow, Poland

**Keywords:** Analogy, Reasoning, Mapping, Distraction

## Abstract

Reasoning by analogy requires mapping relational correspondence between two situations to transfer information from the more familiar (source) to the less familiar situation (target). However, the presence of distractors may lead to invalid conclusions based on semantic or perceptual similarities instead of on relational correspondence. To understand the role of distraction in analogy making, we examined semantically rich four-term analogies (A:B::C:?) and scene analogies, as well as semantically lean geometric analogies and the matrix task tapping general reasoning. We examined (a) what types of lures were most distracting, (b) how the two semantically rich analogy tasks were related, and (c) how much variance in the scores could be attributed to general reasoning ability. We observed that (a) in four-term analogies the distractors semantically related to C impacted performance most strongly, as compared to the perceptual, categorical, and relational distractors, but the two latter distractor types also mattered; (b) distraction sources in four-term and scene analogies were virtually unrelated; and (c) general reasoning explained the largest part of variance in resistance to distraction. The results suggest that various sources of distraction operate at different stages of analogical reasoning and differently affect specific analogy paradigms.

## Introduction

Analogical reasoning is a core mechanism of human cognition, playing an important role in problem-solving (Gick & Holyoak, 1980; VanLehn, 1998), concept learning (Goldstone & Medin, 1994), language (Gentner & Kurtz, 2005; Lakoff & Johnson, 1980), and other cognitive functions. To make a valid analogy, one must identify relational correspondence between two situations and use it to transfer the knowledge from a better-known situation (source) to a less familiar one (target) (Holyoak, 2012).

A dominant theory explaining analogical reasoning is the structure-mapping theory formulated by Gentner (1983). It posits mapping as a key and indispensable stage. Mapping consists of two steps: alignment and inference. First, reasoners must identify and align the structure of relations between two situations (they need to match the corresponding relations and arguments). Second, they need to infer the missing knowledge in some relations and the arguments in the target situation using the corresponding relations and arguments in the source situation. Finally, they evaluate the validity of their inference.

However, in some conditions people are less likely to base their mapping on the (“deep”) correspondence between relations and instead tend to follow (often incorrectly) the (“surface”) similarity between the objects in the two situations, such as when making an analogy under time pressure (Goldstone & Medin, 1994) and along with a parallel task (Waltz et al., 2000) as well as when the analogy is highly complex (Chuderska & Chuderski, 2014; Krawczyk et al., 2014).

On the grounds of the structure mapping theory, Gentner and Smith (2012) distinguished three key factors influencing how mapping proceeds. One factor is a principle called *structural consistency*, a preference for “one-to-one” alignment, as analogy making is easier when the reasoner can match each object from the source with one and only one object from the target. Another factor is a principle called *systematicity*, a preference for comprehensive correspondence that comprises as many relations and arguments as possible in a given situation. More systematic mapping typically leads to more valid inferences. A number of authors have supported the role of both structural consistency and systematicity in mapping (Clement & Gentner, 1991; Markman & Gentner, 2000; Gentner & Toupin, 1986; Krawczyk et al., 2005).

The third factor, which has been relatively less studied in mentally healthy adults, and which definitely requires better understanding, is *transparency*. It is described as a degree of surface similarity between relationally corresponding objects. In transparent analogies, the corresponding objects are identical or at least semantically and/or perceptually highly similar, while objects that play different roles are highly dissimilar. This makes mapping relatively easy as the objects’ perceptual and semantic characteristics can guide the thinking process (e.g., ketchup comes from tomatoes as cider comes from apples). In less transparent analogies, corresponding objects are dissimilar – such as belonging to distant semantic categories, so structural alignment must rely only on the relations among the objects and as a result is more difficult (cider comes from apples as milk comes from cows). Furthermore, invoking relationally unrelated but similar objects can pose a certain level of distraction for participants. The extreme case of low transparency imposing strong distraction during mapping consists of cross-mapping, in which highly similar or even identical objects play different roles within the relational structure (e.g., Chuderska & Chuderski, 2014; Gentner & Smith, 2012; Gentner & Toupin, 1986). For instance, when a boy is chasing a cat in one scene, and a dog is chasing a boy in another scene, the two boys cannot be matched together in the course of analogical reasoning, because they play different relational roles (a subject vs. an object of an action).

In this study, we aimed to better understand the role of various types of distraction in adult analogy making. Crucially, as to date virtually all studies have applied a single analogy task, we investigated whether the specific effects of distraction could be generalized across two analogy paradigms, namely pictorial four-term analogies and scene analogies. In the former paradigm, we presented distinct categories of distractors as incorrect response options (ranging from purely superficial matches to those resembling the deep match), and we analyzed their effects in the participants’ responses. We investigated cross mapping in the latter paradigm. We asked how strongly the individual resistance to distraction correlates between these two tasks. Additionally, geometric analogies in which no explicit distraction was present were administered to evaluate to what extent analogical reasoning ability in non-semantic task can explain resistance to distraction.

### Paradigms to investigate analogical reasoning

Due to their framing (typically non-verbal problems, including familiar situations and objects) and ecological validity (resemblance to making real-life analogies), four-term analogies and scene analogies have been highly useful for studies on healthy adults (e.g., Chuderska & Chuderski, 2014; Waltz et al., 2000) and children (e.g., Richland et al., 2006; Richland & Burchinal, 2013; Thibaut et al., 2010), as well as in various clinical groups (e.g., Krawczyk et al., 2008, 2010, 2014; Kucwaj & Chuderski, 2020; Morrison et al., 2004). Both paradigms are well suited for introducing precise experimental manipulations aimed at uncovering mechanisms of distraction in analogy. In contrast, geometric analogies are devoid of semantic content and require finding and matching geometric transformations among the objects, loading strongly working memory and analogical reasoning ability (Chuderski, 2015).

#### Four-term analogy paradigm

Pictorial four-term analogies, also known as proportional or propositional analogies, have a simple form “A:B::C:D?” The relation linking A and B needs to also apply to C and D. For example, for the abovementioned analogy *apple:cider::cow:milk*, the relation is *the origin of a product*. The respondent should select object D (*milk*) from a set of possible response options. In this task, only part of the mapping process takes place. The reasoner must only abstract the relation as the arguments and the alignment structure are already provided. Therefore, the four-term task seems to load working memory to a lesser extent, as compared to the scene analogy task, in which the entire alignment structure (or at least its part) needs to be identified.

The question of how people process the A, B, and C terms as well as the response options while solving four-term analogies has become an important issue in recent eye-tracking research on different age groups (Starr et al., 2018; Thibaut & French, 2016; Vendetti et al., 2017). Two potential strategies were proposed on the basis of leading computational models of mapping: the projection-first strategy and the alignment-first (or structure-mapping) strategy (Gentner & Forbus, 2011). In the projection-first strategy, the reasoner begins by projecting information from the base to the target (see Hummel & Holyoak, 1997). In the case of four-term analogies, this strategy would be reflected in that people first aim to establish the relation between A and B (base pair) and then analyze C to find the D with the same relation. In contrast, in the structure-mapping strategy, the alignment of items composing the base and the target occurs first (see Gentner, 1983; Gentner & Forbus, 2011; Markman & Gentner, 1993). In the case of four-term analogies, this strategy would be reflected in first processing A and C, rather than A and B. The second step would be to align B with the target. Analyses of saccadic trajectories have suggested that most adults apply the projection-first strategy, focusing on the A-B pair at the beginning of a trial, and then switching their attention to C (Starr et al., 2018; Thibaut & French, 2016; Vendetti et al., 2017). However, using a strategy classification algorithm, Vendetti et al. (2017) reported that some reasoners still apply the alignment-first strategy on some occasions, but less frequently (307 trials recognized by the algorithm as the alignment-first strategy vs. 446 trials recognized as the projection-first strategy).

Thibaut and French (2016) investigated a third possible strategy, which was deduced from the relational-priming model (Leech et al., 2008). The model posits that once a reasoner has determined the A-B relation, priming would automatically lead to find the target that matches C, without paying attention to the other options. Nevertheless, the data did not support this hypothesis in young children. Instead, children used another strategy: They seemed to organize their processing around C, paying little attention to the A-B relation. Thibaut and French (2016) suggested that children may focus on the overarching goal – to find the picture that goes with C – and, as a consequence, they omit the sub-goal to analyze the first pair. Interestingly, researchers can draw children away from this strategy by guiding their attention to the source pair at the beginning of a trial (Glady et al., 2017). Starr et al. (2018) and Vendetti et al. (2017) further investigated this kind of processing, called the semantic-constraint strategy, which assumes that people prioritize C over the A-B pair in order to narrow the search space, since A and B might be related in multiple ways. Vendetti et al. (2017) found that the semantic-constraint strategy yielded the most error-prone processing, as compared to the project-first strategy (correlating positively with accuracy) and the structure-mapping strategy (unrelated to accuracy).

One of the crucial factors that affects solving four-term analogies is the presence of distractors among the response options (e.g., Krawczyk et al., 2008; Kucwaj & Chuderski, 2020; Thibaut et al., 2010). In the most typical version of distraction condition, the response options comprise the correct option as well as either a semantic distractor and two unrelated objects (e.g., Glady et al., 2017; Krawczyk et al., 2008; Thibaut & French, 2016), or a semantic distractor, a perceptual distractor, and one unrelated object (e.g., Starr et al., 2018; Whitaker et al., 2018; Vendetti et al., 2017). A semantic distractor is defined as an object that belongs to the same semantic category as C (Krawczyk et al., 2008) or as an object whose meaning is associated with C (Starr et al., 2018; Vendetti et al., 2017). The perceptual distractor is an object perceptually similar to C (Krawczyk et al., 2008), typically sharing the same color and shape (Starr at al., 2008; Vendetti et al., 2017). The distractors were shown to hinder performance of patients with frontotemporal lobar degeneration, while healthy controls were able to ignore the distractors (Krawczyk et al., 2008). Also, children were prone to choose the distractors, with semantically related distractors being more difficult for them to reject than were perceptual distractors. Additionally, children’s failures on response-inhibition tests were positively correlated with proneness to distraction in analogies.

However, the existing research on the role of distractors has not been free from certain methodological issues. Crucially, in semantically rich tasks, it is difficult to design response options that perfectly match the above definitions. Sometimes objects that were originally defined as perceptual distractors combine characteristics of both perceptual and semantic distractors. For instance, in the Krawczyk et al. study (Krawczyk et al., 2008), the perceptual distractor to a hammer was a gavel. Actually, these two objects have more in common than just perceptual appearance as both are used to hit things, they make similar sounds, and they can be held in the same way, etc. In some languages (e.g., French, Portuguese, Polish), a single word represents both objects. Therefore, for the reasoner to precisely differentiate the distractors, a third type of distractor is needed: a categorical distractor, which is defined as an object related to C both semantically and perceptually.

Furthermore, semantic, categorical, and perceptual distractors altogether are not exhaustive of all the potential sources of distraction. Consider a four-term analogy in which a r​ose (A) relates to the sun (B) (acquires energy from), and a rabbit (C) relates to another object (D) according to an analogous relation. A given responder might identify the being-the-food relation correctly, but at the stage of selecting the argument, they might become distracted by an object that is edible but not typical for rabbit consumption (e.g., a hot dog). Another example is paper:wood::wool:? Someone might think of livestock, but instead of selecting a sheep as D, they may choose a goat – an animal that humans breed, but not for wool. To our knowledge, no one has yet examined such relational distractors, and their introduction into the four-term analogies may help to test whether late stages of reasoning (the application of an already-identified relation) are also prone to distraction. Responders’ frequent selection of this option would suggest that even after identifying the key relation, they might still commit errors.

#### Scene analogy paradigm

In a scene analogy task, a participant is provided with two pictures (scenes). In the source scene, one object is highlighted and the participants need to identify a corresponding object in the target scene. To complete the task, participants need to identify the relations and their arguments in the source scene and then validly map them onto the target scene (see Markman & Gentner, 1993). The level of difficulty of scene analogies varies depending on the relational complexity (the number of arguments involved in a relation), the degree of transparency, and the presence or absence of living objects (e.g., Chuderska & Chuderski, 2014; Krawczyk et al., 2014; Morrison et al., 2011). Studies have shown that young children are especially vulnerable to low transparency, as reflected by cross-mapping (Gentner & Toupin, 1986). However, even healthy adults have been impacted when similar objects playing different relational roles are introduced in the target scene. For instance, Chuderska and Chuderski (2014) reported a strong drop (*η2* = .57) of accuracy in the cross-mapping condition (*M* = .52), as compared to a non-cross-mapping condition (*M* = .66).

Interestingly, Krawczyk et al. (2014) did not observe the effect of distraction, neither in healthy participants nor in clinical groups, specifically schizophrenia patients and people with autism spectrum disorder. The lack of a distraction effect, especially in schizophrenia patients, was surprising considering the profound cognitive deficits related to the diagnosis (see Bowie & Harvey, 2006). Presumably, this counterintuitive result might be explained by the distractor condition in applied scene analogies, which were originally developed by Richland et al. (2006) to investigate the effects of relational complexity and featural distraction in children’s reasoning abilities. In the distraction condition, an object or an actor playing an important role in a relation in the source scene was also present in the target scene. However, a distracting object/actor was placed outside the key relation, playing no active role in the target scene (e.g., a dog chasing a cat in the first scene, and a dog lying on the grass in the target scene in which a boy is chasing a girl). Apparently, reasoners can easily ignore distractors not involved in the relation during the analogical reasoning process, even if they suffer from cognitive deficits. Perhaps, only the extreme case of low transparency represented by the cross-mapping condition – in which the distractor plays different relational roles in both scenes, as it did in Chuderska and Chuderski’s study (2014) – may evoke distraction in adult participants. Nevertheless, data on the cross-mapping effect in the adult population are limited.

#### Semantically lean analogy paradigm

The most popular version of a semantically lean analogy paradigm includes geometric analogies. Geometric analogies take the form of either a four-term or a series completion task. As they contain only the geometric shapes, the reasoner need only process the syntactic operations (various geometric transformations), and not the semantics, to make the correct analogy. Geometric analogies strongly load working memory, and researchers commonly use them to examine mapping at various levels of complexity and the individual differences therein (e.g., Bethel-Fox et al., 1984). Typically, the geometric analogy tasks contain no explicit distractor options. Rather, the response options vary in similarity to the target, depending on the number of correct transformations they share with it. Geometric analogies can be considered a relatively purer method to investigate analogical reasoning per se, as neither specific domain knowledge nor vocabulary are required to solve them.

### Research questions

To improve the understanding of the role of distraction in analogical reasoning, we applied a novel variant of the pictorial four-term analogy task, in which we extended the number of distractor categories to four: semantic, categorical, perceptual, and relational distractors (for their description see the section *Four-term analogies* and Table 1). Furthermore, unlike previous studies, we introduced to the four-term task distracting response options related to the B term. Focusing solely on the C-related distractors, as has been done up until now, has been a limitation as this kind of manipulation might have been ineffective with regard to people following the structure-mapping (alignment-first) strategy, in which response selection might be primarily affected by the B term (aligning it with the target). By comparing the selection frequency of the B- versus the C-related distractors, we attempted to examine to what extent the participants exploit information provided by object B during the mapping process. If they strongly rely on B, they should be prone to B-related distractors; otherwise, such distractors should have little effect. We expected that the extended range of response choices may be able to provide additional support to the eye-tracking studies’ results, which show that the project-first strategy (matching A with B and then C with D) is more common among adults than is the structure-mapping strategy (matching A with C and looking for a D option that would match B). Therefore, the first research question was this: “Which distractor categories most strongly divert the participants from the correct analogical reasoning process?”

**Table 1:** Definitions of each distractor type included in the four-term analogies

Distractor type	Definition of an object
Perceptual	shares similar shape and color to B/C (e.g., tomato and red ball)
Semantic	is associated with B/C in terms of shared domain or occurrence; is dissimilar in terms of perceptual features such as shape and color (e.g., fishing rod is a semantic distractor to fish)
Categorical	belongs to the same semantic category and has similar (but not identical) shape or color (e.g., shark as a categorical distractor to fish)
Relational	could potentially constitute an argument for the relation linking A-B and C-D, but not when the specific object (C) plays the role in a relation (e.g. a hot dog as an object that could fulfil the relation source of energy but not when a rabbit plays the role of an agent in the analogy rose:sun::rabbit:?)

Obtaining the answer to this question can shed light on the stage at which analogy-making is the most strongly prone to distraction. Selecting perceptual distractors reflects the most superficial aspect of analogy processing, while semantic and relational distractors suggest a deeper level of such processing. Considering that we studied a group of mentally healthy adults from 18 to 31 years old, we expected the prevalence of semantic distractors among incorrect responses, with a very rare selection of perceptual distractors (i.e., completely irrelevant responses). Introduction of categorical and relational distractors was more exploratory, since the former is far less popular in four-term analogies, as compared to semantic and perceptual lures, and no scholars have, to our knowledge, examined the latter in studies of four-term analogies.

Furthermore, we applied the scene analogy task in the cross-mapping condition to investigate the relationship of distraction in the two hallmark analogy paradigms. Scene analogies and four-term analogies are both intuitive – their instruction is easy to follow and they include based on the familiar content of everyday situations and objects. Nevertheless, the course of individuals’ analogical reasoning process seems to vary between the two paradigms, crucially at the mapping stage. In scene analogies, the comprehensive mapping of relations and their arguments is essential, especially under cross-mapping. In contrast, in the four-term analogies, the alignment structure and the arguments are already provided, so the reasoners have no need to extract them from a broader context and run the full-blown mapping. The application of both tasks, to date rarely studied jointly, will allow researchers to assess to what extent these two tasks rely on common mechanisms. Consequently, the second research question was: “Would the proneness to distraction in the two analogy tasks be intercorrelated, suggesting a general tendency to rely on non-relational (e.g., semantic, perceptual) aspects of information? Or, perhaps the two tasks would be unrelated, implying that the mechanisms used to cope with the distraction during analogy are rather task-specific?”

Finally, the third research question was this: “What is the relation between resistance to distraction during analogical reasoning and individual differences in more general reasoning factor(s)?” To answer this question, in addition to four-term analogies and scene analogies, we applied semantically lean geometric analogies as a marker of analogy reasoning ability. We also administered the matrix reasoning task to assess fluid intelligence. The goal was to investigate how large a part of the interindividual variance in resistance to distraction in semantically rich analogies can be explained by analogical reasoning ability as well as even more general relational reasoning ability, as assessed with tasks devoid of any semantic load. Can resistance to distraction be simply reduced to general reasoning ability (i.e., people who reason effectively can ignore distractors because they easily identify the correct response), or is it independent from reasoning ability (distractors affect even those who reason effectively)? In particular, the lack of correlation between resistance to distraction in analogies and general reasoning ability would suggest that there exists a specific mechanism responsible for solving analogies in the presence of distraction.

In summary, in this study, we introduced several modifications to the hallmark four-term analogy paradigm, which scholars for decades have broadly applied in studies on analogical reasoning. This novel method allows for more fine-grained probing of sources of distraction in analogical reasoning and could potentially be useful for future studies on various samples (e.g., children, clinical groups). Additionally, the present study represents a comprehensive approach to measuring reasoning by analogy by using three different tasks. As the precise impact of distraction on analogical mapping in the healthy adult population remains elusive, the present study has the potential to substantially contribute to knowledge on humans’ ability to reason by analogy.

## Method

### Participants and procedure

A total of 220 volunteers were recruited via the Internet. Participants visited a Central European university’s psychological laboratory and solved the tasks described below in groups of five to nine people. Each person was paid the equivalent of 10 euros in local currency. All signed a written consent form to participate, were screened for normal or corrected-to-normal vision, had no history of neurological disorders, and were informed that they could stop the experiment at any time. All other aspects of the study conformed to the WMA’s Declaration of Helsinki. The four-term analogies and the scene analogies were applied at the very beginning of the procedure, with the order of the two tasks randomized. These were followed by the Raven’s Advanced Progressive Matrices (RAPM) and the geometric analogies. Next, a battery of other cognitive tasks was administered as part of another research project (unrelated to the study herein), and thus we will not describe it further here. In total, ten participants were excluded from the analysis: nine due to their very short mean reaction time (less than 4 s) or an unusual pattern of responding in analogical reasoning tasks (over-representation of otherwise rarely selected options), which suggested random guessing; and one person because of missing data due to technical issues. In the case of one participant, one missing value in the computer version of the geometric analogies was imputed using the mean score. The final sample counted 210 people (143 women, 67 men; age 18–32 years, *M* = 22.9, *SD* = 3.2).

### Four-term analogies

Four-term analogies were computerized. This task consisted of 26 four-term analogy problems. All these items were selected (and, additionally, seven of them were revised) from a larger bank of 40 problems, which had been validated in a sample of 251 young adults (Kucwaj et al., 2021). The remaining 14 items from the bank were discarded, as they yielded either ceiling or floor scores, or led to highly idiosyncratic responses. Each problem had the A:B::C:D? structure, where the responder was to select D from ten options. All stimuli were pictures of common objects and no two pictures were identical within the entire item set. Participants were asked to find an analogy between two pairs of objects according to the rule “A is to B as C is to D.” The following example with an explanation was provided: Milk (A) is to cheese (B) as flour (C) is to bread (D). Participants were told that A, B, and C would be given, and they needed to select D from a set of eight response choices displayed in random order. Participants were instructed that more than one object may seem to go with C but they were to choose the only one that was definitely related to C in the same way that B was related to A. To respond, the participants clicked a picture and confirmed their choice by clicking the “next” button. For each problem, the maximum time for response was 30 seconds. The task was preceded by instruction and two training problems with feedback.

**Fig. 1:**
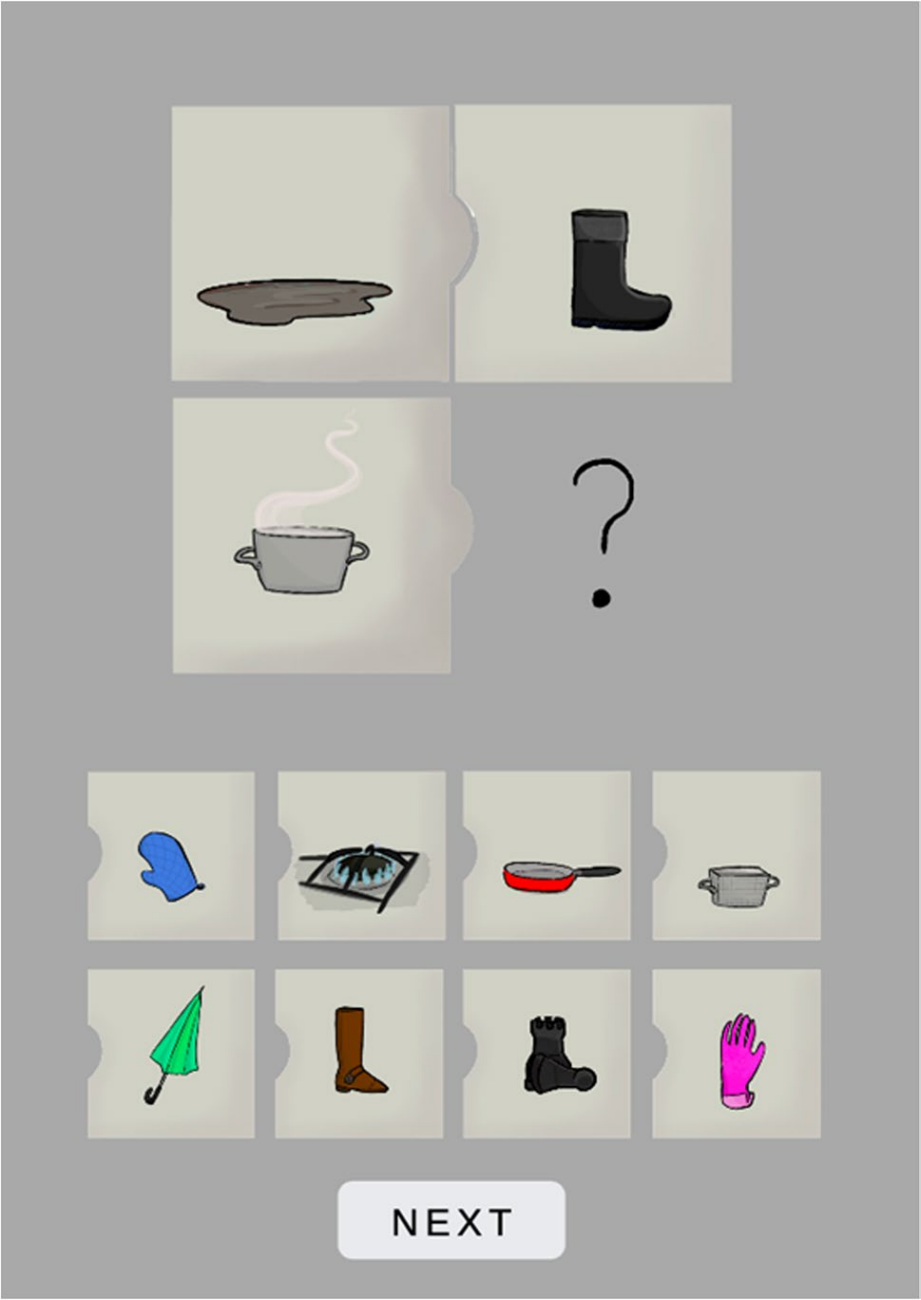
Sample item of the Four-term Analogy. The A:B::C:D? problem (top) to solve: puddle:rain boot::hot pot:? Response options (bottom) are the following: ​an oven mitt is the correct response, a gas burner is a semantic distractor related to C, ​a frying pan is a categorical distractor related to C, a basket is a perceptual distractor related to C, an umbrella is a semantic distractor related to B, a boot is a categorical distractor related to B, a chess pieces are a perceptual distractor related to B, and a rubber glove is a relational distractor. The order of response options was fully random across all trials in the study

The response options included the correct answer, the relational distractor to the correct response, the semantic distractor related to B, the categorical distractor related to B, the perceptual distractor related to B, the semantic distractor related to C, the categorical distractor related to C, and the perceptual distractor related to C. The perceptual distractor was defined as an object that shares a similar shape and color to B/C (e.g., red ball – red tomato). Its selection was interpreted as following the basic featural similarity between the objects and thus a very shallow processing of the analogy. The categorical distractor was defined as an object that belongs to the same category and has a similar, but not identical shape or color to B/C (e.g., green bush – olive tree). The perceptual similarity was weaker in this case than for the perceptual distractor. The choice of the categorical distractor suggests that although the reasoner has ignored or rejected the least rational option (a perceptual lure), they have only analyzed relatively superficial features, which might be sufficient for successful analogical reasoning but only in rare cases. The semantic distractor was an object associated with B/C in terms of a shared domain, function, or occurrence (e.g., a fish and a fishing rod). This type of distractor, when selected, indicated an elaborated search of the semantic memory, beyond a sheer perceptual or categorical match. The relational distractor was defined as an object that could potentially constitute an argument for the relational role in a given analogy, but not when the specific object (C) plays the role in a relation. For example, in the following analogy: a puddle (A) is to a rain boot (B) as a hot pot (C) is to an oven mitt (D) (see Fig. 1), a rubber glove fulfills the correct relation, as it can protect hands but not from high temperatures (it protects from chemicals, dirt etc.). Therefore, relational distractors suggest that the participant identified the target relation to some extent but had not completed the mapping process, or their selection had been disturbed at the very last stage.

### Scene analogies

**Fig. 2:**
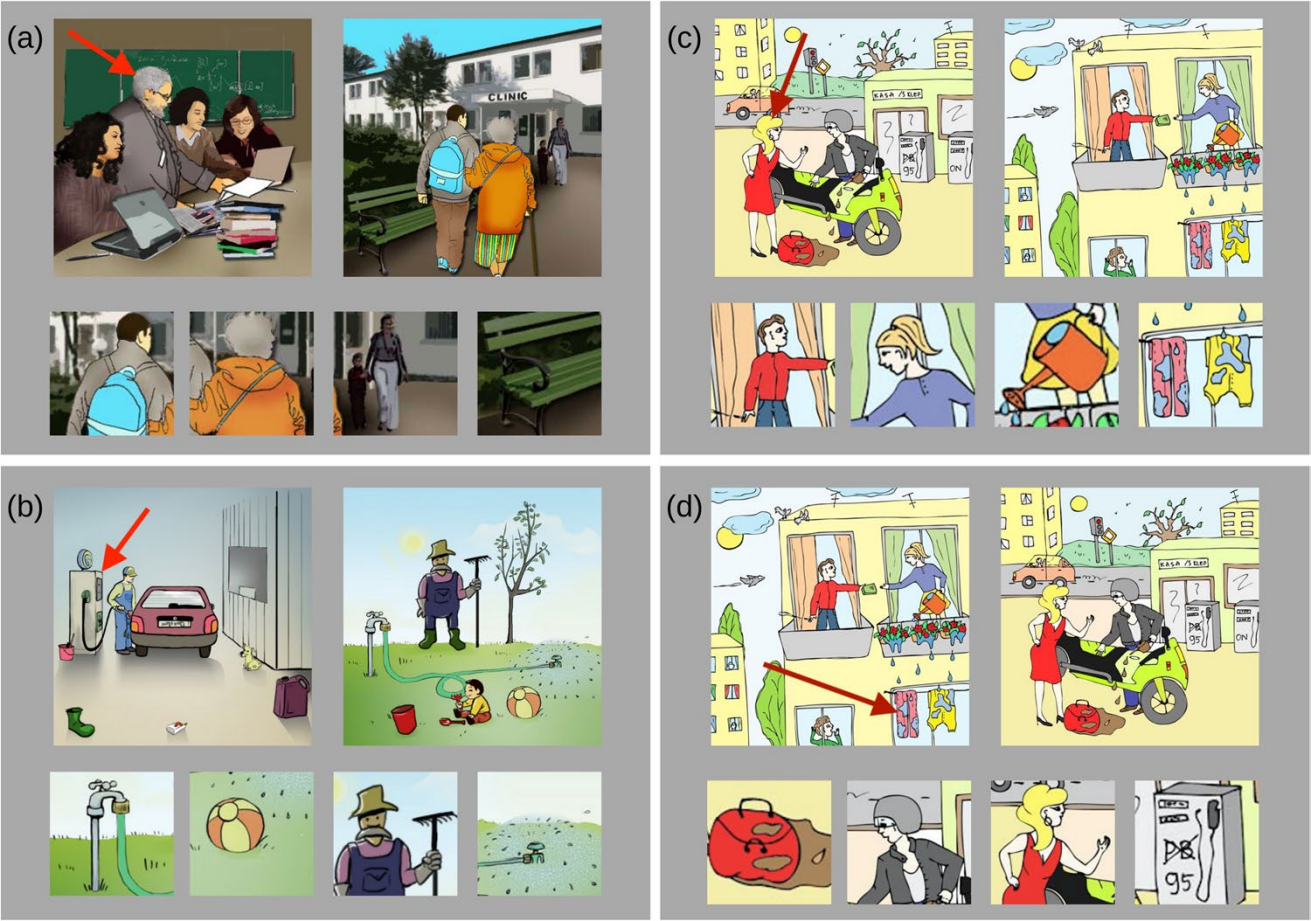
Example items of Scene Analogies. **a** A cross-mapping item. On the left, an elderly man is explaining something to a group of students, so he plays the role of a leader. On the right, an elderly woman is assisted by a young man who plays the role of a leader (so, mapping the two elderly persons together leads to an error). The four response options at the bottom of the panel included the correct response (the young man), a distractor (the elderly woman belonging to the same category of people as the man pointed to in the first scene), and two irrelevant (i.e., non-distractor) objects (a woman passing by with a child and a bench). **b** A non-cross-mapping item. Response options comprise the correct response (an outdoor tap) and three irrelevant (non-distractor) objects. **c** A cross-mapping item. On the left, a woman is distracting a man biker who, as a result, spills gasoline on a bag. On the right, a woman is being distracted by a man neighbor while watering her plants, as a result spilling the liquid on the laundry. The four response options included the correct response (the man), a distractor (the woman) and two irrelevant (i.e., non-distractor) objects (a watering can and the laundry). **d** The c pair is reversed and cross-mapping is absent. Response options comprise the correct response and three irrelevant objects. For all items, the order of response options in the response set was random for each participant

This task consisted of 36 pairs of scenes selected from the problems originally developed by Chuderska and Chuderski (2014), who reported a significant effect of cross-mapping observed in healthy adult subjects. They asked the participants to locate in the first scene an object that was either indicated with a red arrow or marked with a red rim. Then, the participants had to determine the role of this object in the situation depicted. Their task was to analyze the second scene in order to identify the object that plays the same role as the object marked in the first scene. Half of the test items included cross-mapping, which, as noted above, is defined as a situation in which similar objects play different relational roles in both scenes, so mapping them according to their similarity leads to errors (e.g., Chuderska & Chuderski, 2014; Gentner & Smith, 2012; Gentner & Toupin, 1986). Figure 2 presents four examples of Scene Analogies. The first pair of scenes (panel a) belongs to the cross-mapping condition: An elderly man from the first scene plays the role of a tutor/leader to students, while an elderly woman in the second scene is guided/led by a younger man. The second pair (panel b) consists of an example of the non-cross-mapping condition.

In total, the task included four sets of items: (a) 12 pairs of scenes with cross-mapping (Fig. 2a); (b) 12 pairs without cross-mapping (Fig. 2b); as well as (c) another six pairs with cross-mapping (Fig. 2c), which appeared in the task twice as they also served to create (d) another six pairs without cross-mapping (Fig. 2d) by a reverse of the order of the scenes in the pair. (In the scenes d, no object similar to an object indicated with a red arrow in the source scene was present in the target scene.) In total, the task included 18 items with cross-mapping (set a and c) and 18 items without cross-mapping (set b and d). Sets c and d were introduced as “control” items, to check whether the cross-mapping effect occurs for items with the same content, that is, to validate that the thesis effect can be attributed univocally to the similarity between the red-marked object and the distractor object, and not to accidental differences in the specific content between the scenes. The order of the item presentation was the following: three randomly selected items from each of sets c and d (all six pairs being different); 24 items from sets a and b in fully random order; and the six remaining items from sets c and d. The response option structure in the 18 items with cross-mapping fully matched that for the 18 items without cross-mapping in terms of the number of either animate or inanimate objects as well as the number of objects that did or did not play a role in the relation. The task was preceded by two training items with feedback (one item with cross-mapping and one without). The maximum time allowed for each response was 30 s per item. Figure 2 also presents one example from set c and one from set d. In set c, the target scene includes an object that is similar perceptually to an object indicated with an arrow in the source scene and shares the same semantic category with it (cross-mapping). In d, the content is identical to that in c, but the scene order is reversed, and the object indicated with an arrow in the source scene is not similar to any object in the second scene (non-cross-mapping).

### Geometric analogies

**Fig. 3:**
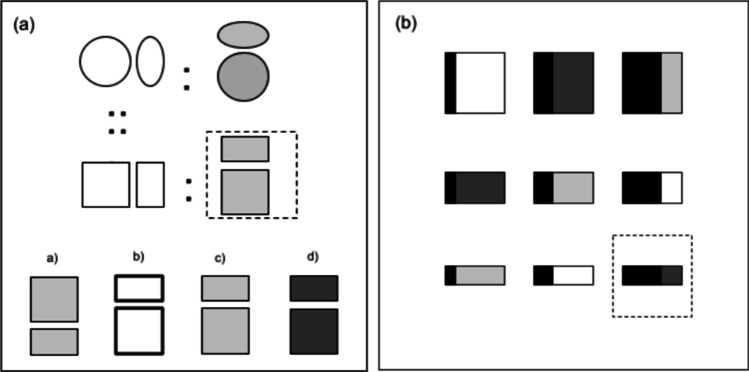
**a** An exemplary item of Geometric Analogies and **b** an RAPM-like item (not an actual item); the correct response (dashed rim) is missing and has to be selected out of four/eight options in the case of Geometric Analogies/RAPM (not depicted in the example)

The task consisted of 36 items in the form A is to B as C is to X, where A, B, and C were relatively simple shape patterns. A and B were mutually related based on the transformations of their perceptual features, such as the shape’s size, color, thickness, etc. The task was to select one pattern out of four that was related to pattern C as B was related to A. One half of the items were on a computer, and the other half were in a paper-and-pencil format. (This manipulation, related to another project, aimed to compare whether the presentation format changed the test validity. It did not.) The time allocated for all items was 30 min. The final score was a sum of correct responses in all items. The task was meant to capture an individual’s analogical reasoning performance (primarily, analogical mapping) irrespective of semantic content. Fig. 3a presents a Geometric Analogies item.

### General reasoning ability

Raven’s Advanced Progressive Matrices (RAPM) consists of 36 items. Each item comprises a 3 × 3 matrix of shape patterns with a missing bottom-right pattern. Among eight response options, one correctly fulfills the gap. The task was to determine the rules that govern the matrix and to apply them correctly to identify the missing pattern. One half of the items were on a computer, and the other half were in a paper-and-pencil format, for the same reason as in the case of Geometric Analogies. The time allowed to solve the entire test was 40 min. The final score was a sum of correct responses in all items. The RAPM score is commonly believed to be a close proxy of general reasoning ability (fluid intelligence, Gf) (McGrew, 2009). Figure 3b presents an example of an RAPM-like item.

## Results

We analyzed the data with StatSoft Inc. STATISTICA version 64 and RStudio version 4.0.3 (vegan package to conduct variance partitioning, Oksanen et al., 2020).

### Distraction in four-term analogies

Dependent variables in Four-term Analogies included the total error rate and the error rate for each distractor type. The total error rate was computed as the proportion of all errors in all trials. The error rate for each distractor type was computed as the proportion of the number of choices of each distractor type in all the errors committed in the task. In the case of both variables, we excluded the trials in which the participants entered no response (14 trials out of 5,460 of all trials in the task).

**Fig. 4:**
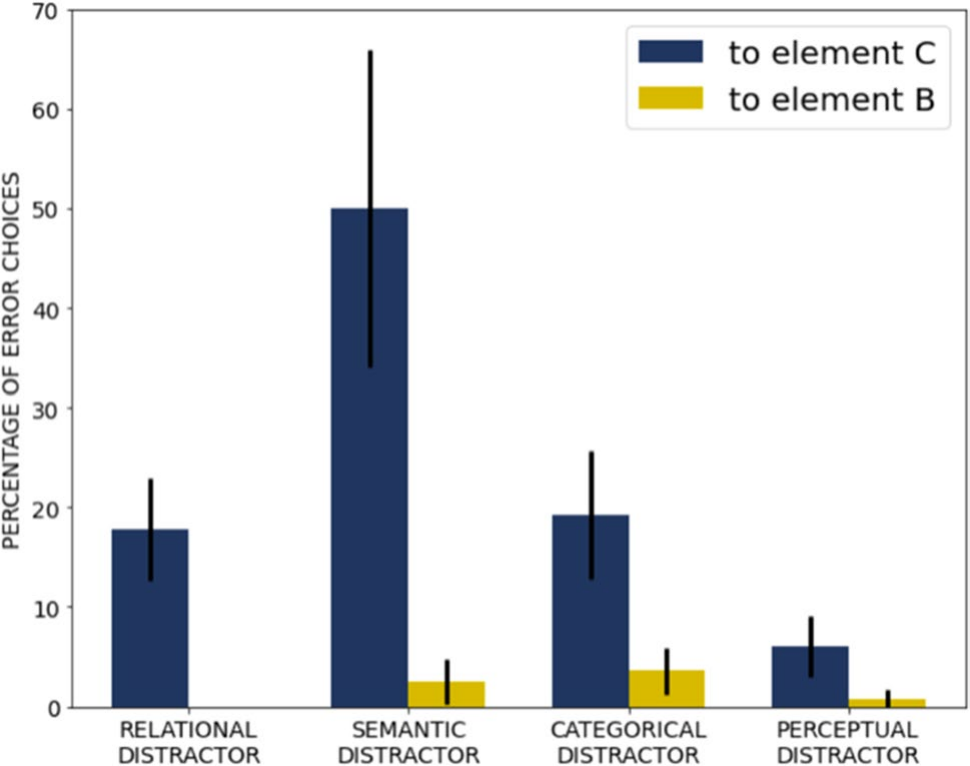
Percentage of error choices in Four-term Analogies, computed as the proportion of the number of times each error option was committed over all errors committed. The bars indicate 95% confidence intervals

The total error rate equaled *M* = 10.33% (*SD* = 11.88%, range 0–72). Figure 4 presents the selection rates for each error option. Participants very rarely selected the distractors related to B (semantic, categorical, perceptual) and the perceptual distractors related to C, making from 1% to 6% of all the errors committed, depending on the distractor type (14% in sum). The semantic, categorical, and relational distractors related to C comprised 50%, 19%, and 18% of all the errors committed, respectively. A Wilcoxon signed-ranks test showed that the participants selected significantly more frequently the semantic distractors related to C than the relational (*Z* = 5.77, *p* < .001) or the categorical distractors (*Z* = 5.79, *p* < .001). They chose the relational distractors as frequently as the categorical distractors (*Z* = 0.47, *p* = .637).

### Cross-mapping in scene analogies

The dependent variables in Scene Analogies were the total error rate (the proportion of incorrect responses in all 18 trials of the cross-mapping and the non-cross-mapping condition) and the distractor error rate (the number of cross-mapping trials in which a distractor was chosen; obviously, this variable was not applicable to the non-cross-mapping condition). We excluded 30 out of 1,258 incorrect trials in the cross-mapping condition due to non-response. The total error rate in items without cross-mapping equaled *M* = 14.63% (*SD* = 13.1%). With cross-mapping, it equaled *M* = 33.28% (*SD* = 17.5%), and the difference was highly significant, *t*(209) = 17.38, *p* < .001, Cohen’s *d* = 1.2. The majority of the errors in the latter condition (75% errors) consisted of the distractor errors.

Looking more closely at the particular item sets, the cross-mapping effect was highly significant both for the unique items (set a vs. set b), *t*(209) = 12.58; *p* < .001, and for the repeated-reversed items (i.e., set c vs. set d), *t*(209) = 14.83, *p* < .001. The cross-mapping effect in items sharing the same content (i.e., c vs. d), which matched the effect in the items differing in content (i.e., a vs. b), indicated that this effect can be attributed univocally to the presence of distractors, and not to accidental differences in the items’ content.

### Geometric analogies and raven’s advanced progressive matrices (RAPM)

The dependent variables in Geometric Analogies and RAPM were the error rates computed as the proportion of all errors in all 36 trials. The error rate in Geometric Analogies equaled *M* = 29.7% (*SD* = 15.18%, range 0–86), and in RAPM it equaled *M* = 35.67% (*SD* = 15.7%, range 3–92).

### Relationship between the three analogical tasks and RAPM

The following dependent variables were correlated: the total error rate for Four-term Analogies (note that each error consisted of selecting a distractor); the distractor error rate for the Scene Analogies (i.e., only errors resulting from selecting a distractor in the cross-mapping trials); as well as the error rates in Geometric Analogies and in RAPM (the two tests that did not include any intended distraction). The Pearson correlation for Four-term Analogies and Scene Analogies, that is, the two semantically rich tasks, equaled *r* = .381, *p* < .001. Geometric Analogies and RAPM correlated strongly at *r* = .743, *p* < .001. Geometric Analogies correlated with Four-term Analogies, *r* = .568, *p* < .001, as well as with Scene Analogies, *r* = .373, *p* < .001. The RAPM score correlated just as strongly, *r* = .561, *r* = .434, respectively, both *ps* < .001.

### Variance partitioning

As all four tasks in the study were mutually correlated, sharing a substantial proportion of common variance, in order to estimate the exact contribution to the error rate in Four-term Analogies of the three remaining variables, we applied variance partitioning (see Chuah & Maybery, 1999). In general, this method is applied to the results of multiple regression models in order to estimate the part of variance in a predicted variable that can be attributed to each predictor separately, as well as to the part of that variance that more than one predictor jointly contributes to (that cannot be attributed univocally to a single predictor). Specifically, in the case of two predictors A and B, the part of the predicted variance attributed uniquely to predictor A is estimated as the total variance explained by the model (i.e., *R*^*2*^ it yields) minus *R*^*2*^ of the linear regression model including only predictor B (A = A&B — B). The respective variance attributed uniquely to predictor B is estimated as *R*^*2*^ of the multiple regression model minus *R*^*2*^ of the linear regression model including only predictor A (B = A&B — A). The predicted variance that A and B jointly contribute to equals *R*^*2*^ of the multiple regression model minus the parts of the predicted variance attributed uniquely to A and to B (A+B = A&B — A — B). The calculations in the case of more than three predictors proceeded analogously.

**Fig. 5:**
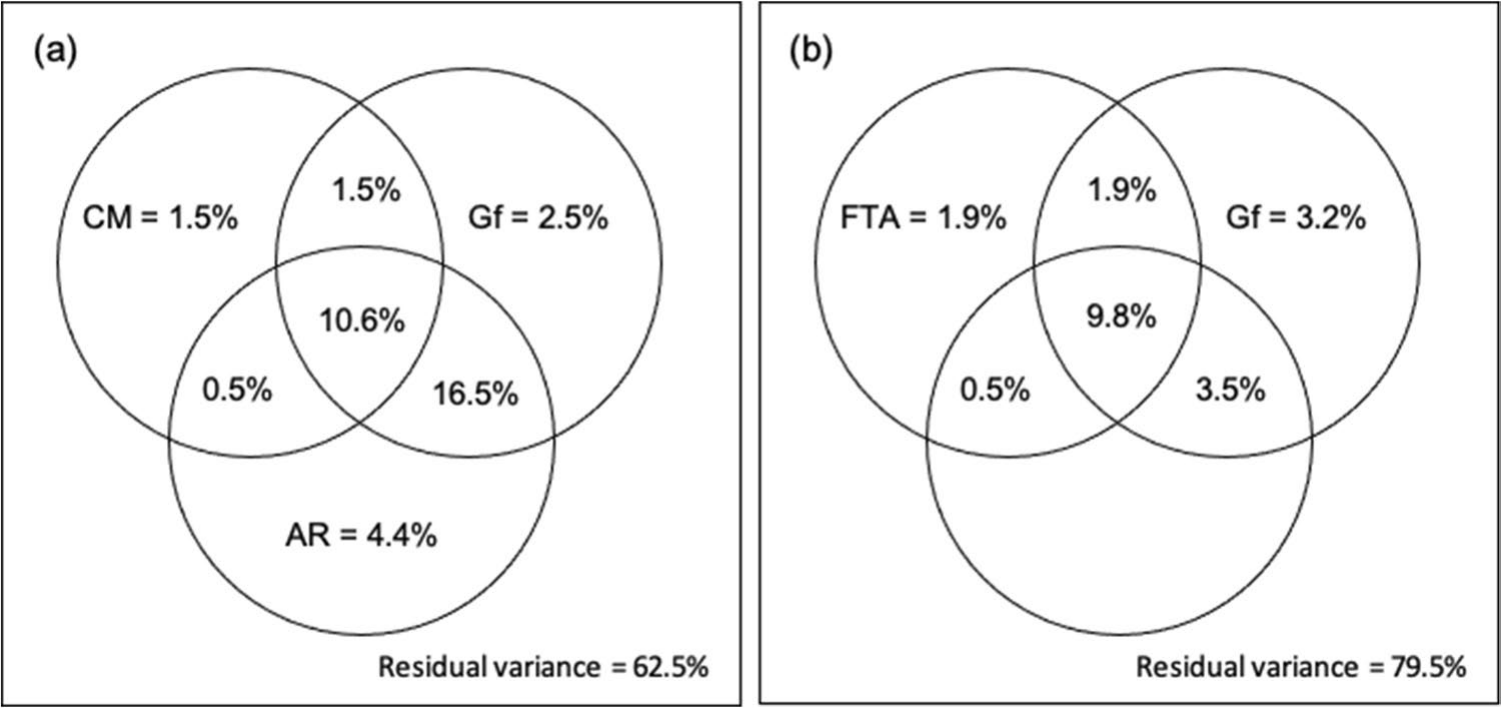
**a** A Venn diagram showing the amount of variance in the total error rate on Four-term Analogies (FTA) predicted uniquely and jointly by the distractor error rate in Scene Analogies (CM), Geometric Analogies (AR), and Raven’s Advanced Progressive Matrices (Gf). **b** An analogous diagram showing the amount of variance in CM predicted uniquely and jointly by FTA, AR, and Gf. Null values are not shown. The numbers representing the amounts of variance were calculated using the variance partitioning method based on the *R*^*2*^ values of the regression models presented in Table 2. See text for description

First, we estimated the amounts of variance in the total error rate in Four-term Analogies that had been predicted separately by the distractor error rate in Scene Analogies’ cross-mapping condition (cross-mapping; CM), the error rate in Geometric Analogies (analogical reasoning; AR), and the error rate in RAPM (general fluid intelligence; Gf), as well as amounts that had been predicted jointly by two (CM&AR, CM&Gf, AR&Gf) and by all the three predictors (CM&AR&Gf) (see Table 2 for the respective *R*^*2*^ values). Next, we calculated each predictor’s unique as well as its joint contribution to Four-term Analogies (FTA) variance. For example, the part of the FTA variance attributed uniquely to CM equaled the amount of the FTA variance predicted by all three variables (CM&Gf&AR) minus the amount of the FTA variance predicted jointly by AR and Gf (AR&Gf). The results of the variance partitioning are presented graphically in Fig. 5a. Out of the 37.5% of the FTA variance accounted for by all three predictors in the multiple regression model, CM contributed uniquely to 1.5% of that variance, AR to 4.4%, and Gf to 2.5%. The joint contribution of all predictors amounted to a total of 10.6% of the FTA variance accounted for. Gf and AR jointly predicted the largest amount of the latter variance (16.5%), whereas the joint contributions of CM and AR as well as of CM and Gf were smaller (0.5% and 1.5%, respectively).

**Table 2:** The amount of variance (*R*^*2*^) in the total error rate on Four-term Analogies (FTA; middle column) and in the distractor error rate on Scene Analogies (CM; right column) accounted for by the regression models including all the possible combinations of predictors (AR = Geometric Analogies, Gf = RAPM)

Predictor(s) for FTA	Predicted FTA variance	Predictor(s) for CM	Predicted CM variance
CM	.141	FTA	.141
AR	.320	AR	.135
Gf	.311	Gf	.184
CM&AR	.350	FTA&AR	.174
CM&Gf	.331	FTA&Gf	.208
AR&Gf	.360	AR&Gf	.186
CM&AR&Gf	.375	FTA&AR&Gf	.205

An analogous variance partitioning analysis concerned the contributions of FTA, AR, and Gf to the distractor error rate in Scene Analogies (CM). The amount of CM variance predicted separately by FTA, AR, and Gf, as well as the amount predicted jointly by pairs of variables (FTA&AR, FTA&Gf, AR&Gf) and by all three variables (FTA&AR&Gf) are presented in Table 2. The results of the variance partitioning are shown graphically in Fig. 5b. Overall, as much as 20.5% of CM variance could be accounted for by the multiple regression model. FTA contributed uniquely to 1.9% of the variance predicted and Gf contributed to 3.5%. The joint contribution of all three predictors amounted to a total of 9.8% of the CM variance accounted for. Again, AR and Gf (3.5%) jointly predicted a relatively large amount of CM variance, Gf and FTA jointly predicted 1.9%, and AR and FTA only predicted 0.5%.

## General discussion

In this study, we comprehensively explored sources of distraction during analogical reasoning using a novel variant of the four-term analogy task. We additionally applied a variant of scene analogies with cross-mapping distraction to investigate the relationship between these two popular analogical paradigms. Additionally, we used two semantically lean tasks involving relational reasoning to test whether the individual differences in general reasoning abilities could predict resistance to distraction in Four-term Analogies and Scene Analogies.

### Which types of distractors bother us most strongly?

The first objective was to identify the strongest types of distractors to analogical reasoning in Four-term Analogies. Specifically, besides the perceptual, categorical, and semantic distractors to the term C that have been studied to date, we introduced relational distractors into this task for the first time. We examined the potentially distracting impact of objects related to B, which, to our knowledge, no one has ever tested before, and found that participants were most strongly misled by the semantic distractors related to C, which error type comprised 50% of all of the errors. The participants were almost three times less likely to choose the categorical distractors related to C (19%) and the relational distractors (18%). They were generally immune to the perceptual distractors related to C as well as to any of the distractors related to B, regardless of their type (semantic, categorical, or perceptual).

These results clearly indicate that the term C comprises the most important object of reference in the response-selection process. On this basis, we conclude that adults rarely apply the structure-mapping strategy according to which A is aligned with C first, and then B is aligned with the target. If they had applied this strategy, we would have observed the distractors to B selected more frequently than our data represents (semantic, categorical, and perceptual distractors related to B comprised only 8% of all of the errors). Instead, the use of projection-first strategy is much more probable (distractors to C comprised 75% of all the errors). Our results are in line with existing eye-tracking research suggesting that the projection-first strategy is the most widely applied strategy by adults (Starr et al., 2018; Thibaut & French, 2016; Vendetti et al., 2017).

We expected the rare selection of perceptual distractors (i.e., the worst options), considering that the studied group consisted of high-performing young adults. Selection of objects perceptually related to B/C would suggest very shallow processing and the lack of comprehension of relational structures, which to our knowledge has only been reported in young children and clinical groups (e.g., Krawczyk et al., 2008; Starr et al., 2018). In contrast, the most frequently selected distractors in the present study consisted of objects semantically related to C. Choosing a semantic distractor for the analogy-making process seems to be more representative of real-life actions than does selecting any other distractor, since efficient reasoning by analogy in real life often requires an extensive search of semantic memory. To commit such an error, one must look for a response beyond sheer perceptual or categorical similarity. Categorical distractors, which are slightly more perceptually similar to C/B and which belong to the same semantic category, are located somewhere between the semantic and the perceptual distractors in terms of the degree of complexity of the information involved in processing. Accordingly, the categorical distractors doubled the perceptual distractors, but amounted to half of the semantic distractors.

The participants selected relational distractors relatively frequently (18% of all errors), which suggests that in some cases the analogical reasoning process might have been disturbed at the very last stage. Even when the task provided the alignment structure, and the participants could easily identify the relation, there was no guarantee that they would succeed with the process of analogy making because the distractor to the target option was still competing for attention. Selecting this distractor most likely indicated their applying the wrong argument for the correct relation.

Nevertheless, it should be noted that the role of relational distractors needs to be interpreted with caution. This type of distractor was less homogenous and not as strictly defined as the other distractors. Still, its 18% selection rate suggests that relational distractors had a significant influence on the effectiveness of the participants’ reasoning. We have not seen this finding reported before.

### How specific is coping with distraction during analogy making?

The second objective, which came about when we applied cross-mapping, was to investigate the relation between the effects of distraction in the two hallmark analogical reasoning paradigms – four-term analogies and scene analogies. In answer to the question “Is the proneness or resistance to distraction shared between these paradigms, or is it, rather, task-specific?” the zero-order correlation between these two measures was moderate (14.1% of variance shared), suggesting initially that distraction in our Four-term Analogies and Scene Analogies might affect some overlapping mechanisms. However, when we precisely tracked all sources of variance using the variance partitioning method, the uniquely shared variance between Four-term Analogies and Scene Analogies appeared to be small (around 2%). This result suggests that the participants were processing the two types of distraction using relatively separate mechanisms, and the moderate relationship initially observed between the two tasks was in fact driven by the variance shared with more general reasoning abilities.

Specifically, as Four-term Analogies and Scene Analogies required the application of substantially different reasoning strategies, distractors could impact a distinct reasoning stage in each task. While the full mapping is essential in Scene Analogies, the structural correspondence is already provided in the problem Four-term Analogies, so the mapping involves only the transfer and not alignment. Secondly, before the mapping takes place in Scene Analogies, the reasoner needs to comprehend the two scenes. Very often the reasoner needs to grasp the context, the social interaction, and the causal relations between the agents in a scene in order to draw the valid conclusion. By contrast, in Four-term Analogies, each problem consists of isolated objects, and the relations between them typically are familiar and functional (e.g., “food,” “habitat,” “tool”). Presumably, in four-term analogies, the reasoner is prone to distraction at the stage of response selection, whereas distractors in scene analogies mainly affect problem comprehension and alignment.

### The role of general reasoning ability for coping with distraction

The third objective concerned the relationship between resistance to distraction during analogical reasoning and more general abilities measured by semantically lean reasoning tasks. The zero-order correlation analysis turned out to be inconclusive, as all the variables were substantially intercorrelated and elicited large multicollinearity. Consequently, we conducted variance partitioning in order to estimate the contribution to the variance in resistance to distraction that was unique to each predictor variable and that was shared by more than one variable.

The results of the variance partitioning suggested that in the case of Four-term Analogies, the largest amount of its variance could be attributed to the two semantically lean tasks (16.5%). This suggests that to cope with distraction in the four-term analogy paradigm, reasoners need to rely on their general reasoning ability to a great extent. The contribution of resistance to distraction in the semantically rich task (the cross-mapping condition) was very small (1.5%), even though the distraction came from the task that most strongly resembled the task being explained.

Similar to the distraction imposed by the cross-mapping condition, only 1.9% of its variance could be attributed uniquely to Four-term Analogies. The largest amount of variance could be jointly attributed to all three predictors (9.8%), which finding also suggests the role of more general abilities.

The above results imply that strong resistance to distraction is, in the first place, a result of effective reasoning (general ability to identify the correct options in a reasoning task). By contrast, the evidence did not indicate the presence of a shared mechanism responsible for coping with distraction in both Four-term Analogies and Scene Analogies. As discussed above, this fact might result from different sources of distraction affecting the processing in each task, with distraction impacted later stages of processing in the former task and earlier stages in the latter task.

Low reasoning ability can lead to a reasoner’s selecting a distractor (especially a semantic and perceptual distractor in Four-term Analogies, or a cross-mapped object in Scene Analogies) in at least two different ways. First, while analyzing the distractors, the participants may have formed a fallback strategy when the they failed to identify the correct relational structure in a given problem (see Bethel-Fox et al., 1984; Vigneau et al., 2006). This would be a problem-dependent strategy, one that is applied only in some problems (especially in very difficult ones). Second, a reasoner who is particularly low in reasoning ability and who has a certain history of failures in reasoning tasks might frequently use semantic and/or perceptual cues when selecting responses, expecting little success if trying to think relationally (see Chuderski, 2015; Kunda et al., 2013). However, these two ways may be difficult for researchers to dissociate in experiments.

Relational distractors seem to negatively affect reasoning in different ways. Most likely, in trials ending with such a response, the participants attempted and, to some extent, succeeded in identifying the relational structure, but failed to apply this structure to the response set (they either misinterpreted the key relation or failed to notice that some other options could satisfy it in a better way). Theoretically, this kind of error should be made by people with a certain level of reasoning ability while the semantic and perceptual distractors might be more typical for people with lower ability levels (see Chuderski, 2015). Unfortunately, the overall number of relational distraction errors in this study was too small to verify this conjecture by looking at their correlation with reasoning ability as a difficult pictorial four-term analogy test, yielding more errors, would be required.

These findings might contribute to an important debate on the nature of distraction in analogical reasoning. Is a higher proneness to distraction related to relatively elementary cognitive abilities such as poor inhibitory control, as is often suggested in the case of children (e.g., Morrison et al., 2006) and clinical groups (Krawczyk et al., 2008; Kucwaj & Chuderski, 2020)? Or is it, rather, tied to difficulties at a more complex level of processing related to problem representation (e.g., problem comprehension and relation abstraction)? We think that elementary cognitive processes seem to play no or at most a very limited role in participants’ selecting a response in Four-term Analogies and in Scene Analogies. The process of solving analogies is long-lasting; therefore, even if a reasoner’s attention has been captured by a distractor due to their poor inhibitory control, the reasoner still has time to verify and correct their choice. More probably, their selection of a distractor is a consequence of failure to comprehend the relation. Considering the results of the variance partitioning, it seems that general reasoning ability is essential for healthy adults’ effective coping with distraction.

### Limitations and future directions

As all of the predictors explained only around 30% and 20% of variance in Four-term Analogies and Scene Analogies, respectively, one limitation of the present study is a possibility that each task (or perhaps particular items in the task) yielded an idiosyncratic source of distraction (e.g., an association between objects reiterated by the media, or an association suggested by the item’s layout). This fact is an unavoidable consequence of applying semantic content, which, in principle, cannot be as precisely controlled as can syntactic material (e.g., geometric analogies). Unfortunately, we cannot easily identify such intrinsic sources of distraction by only observing behavioral responses, as studied here, and more precise measures, such as eye tracking, would be required in the future to identify trials with an unusual fixation distribution. Another possible reason for the substantial amount of unexplained variance is the measurement error. However, the internal consistency of Four-term Analogies and Scene Analogies was satisfactory (Cronbach’s α = .82 and α = .85, respectively), which suggests that in the present study the measurement error was relatively low.

Finally, it should be emphasized that the present conclusions apply only to young healthy adults, who generally cope with Four-term Analogies relatively well (10% error rate). Existing evidence suggests that error rates would be much higher in children and in older adult samples (e.g., Byczewska-Konieczny et al., 2019; Richland et al., 2006; Thibaut & French, 2016), and it is likely that the response patterns would then change (e.g., children would infrequently select relational distractors, but more often consider perceptual distractors). In clinical groups, in which participants’ certain mental faculties are distorted, the distortion may be reflected in their specific error patterns (e.g., a large rate of semantic distractors reflecting distorted semantic processing in schizophrenia patients; Kucwaj & Chuderski, 2020).

## Conclusions

In this study, we asked three questions: “Which distractors would prevail in the hallmark semantic analogy task (four-term analogies)?” “How well would distractibility in this task explain proneness to cross-mapping in another semantic task (scene analogies)?” and “How much variance in scores on these two tasks can be attributed to reasoning ability tapped by the semantically lean tasks?” The results from a large sample of young healthy adults indicated that out of four potential distractor types (relational, semantic, categorial, and perceptual), semantic distractors yielded the strongest impact on four-term analogies (but only when these distractors were associated with the term C), relational and categorial distractors had a mild impact, and perceptual distractors did not matter. However, this distractibility pattern weakly predicted proneness to cross-mapping. Instead, the largest part of variance in the semantic analogies could be explained by reasoning ability, which finding suggests that distractors are selected primarily because the participants failed to represent the underlying relational structure. Overall, the study identified several separable sources of distraction that affect analogical reasoning in young adults. It also validated a novel method of tracking distraction, which in the future can be applied in other groups, both healthy and clinical.
